# Beta-*N*-methylamino-l-alanine: LC-MS/MS Optimization, Screening of Cyanobacterial Strains and Occurrence in Shellfish from Thau, a French Mediterranean Lagoon

**DOI:** 10.3390/md12115441

**Published:** 2014-11-17

**Authors:** Damien Réveillon, Eric Abadie, Véronique Séchet, Luc Brient, Véronique Savar, Michèle Bardouil, Philipp Hess, Zouher Amzil

**Affiliations:** 1 Ifremer (French Research Institute for the Exploitation of the Seas), Phycotoxins Laboratory, rue de l’Ile d’Yeu, BP 21105, F-44311 Nantes, France; E-Mails: Veronique.Sechet@ifremer.fr (V.S.); Veronique.Savar@ifremer.fr (V.S.); Michele.Bardouil@ifremer.fr (M.B.); Philipp.Hess@ifremer.fr (P.H.); Zouher.Amzil@ifremer.fr (Z.A.); 2 Ifremer (French Research Institute for the Exploitation of the Seas), Laboratoire Environnement Ressources du Languedoc Roussillon (LER-LR) F-34203 Sète, France; E-Mail: Eric.Abadie@ifremer.fr; 3 UMR Eco-Bio-Université de Rennes I, F-35042 Rennes, France; E-Mail: Luc.Brient@univ-rennes1.fr

**Keywords:** cyanotoxins, BMAA, DAB, AEG, HILIC-MS/MS, cyanobacteria, bivalve mollusks, French Mediterranean

## Abstract

β-*N*-methylamino-l-alanine (BMAA) is a neurotoxic non-protein amino acid suggested to be involved in neurodegenerative diseases. It was reported to be produced by cyanobacteria, but also found in edible aquatic organisms, thus raising concern of a widespread human exposure. However, the chemical analysis of BMAA and its isomers are controversial, mainly due to the lack of selectivity of the analytical methods. Using factorial design, we have optimized the chromatographic separation of underivatized analogues by a hydrophilic interaction chromatography coupled to tandem mass spectrometry (HILIC-MS/MS) method. A combination of an effective solid phase extraction (SPE) clean-up, appropriate chromatographic resolution and the use of specific mass spectral transitions allowed for the development of a highly selective and sensitive analytical procedure to identify and quantify BMAA and its isomers (in both free and total form) in cyanobacteria and mollusk matrices (LOQ of 0.225 and 0.15 µg/g dry weight, respectively). Ten species of cyanobacteria (six are reported to be BMAA producers) were screened with this method, and neither free nor bound BMAA could be found, while both free and bound DAB were present in almost all samples. Mussels and oysters collected in 2009 in the Thau Lagoon, France, were also screened, and bound BMAA and its two isomers, DAB and AEG, were observed in all samples (from 0.6 to 14.4 µg/g DW), while only several samples contained quantifiable free BMAA.

## 1. Introduction

β-*N*-methylamino-l-alanine (BMAA) is a non-protein amino acid that was discovered in 1967 from the seeds of *Cycas micronesica* on the island of Guam [1]. This neurotoxic compound [2] was suggested to be linked to the high incidence of amyotrophic lateral sclerosis/Parkinsonism-dementia complex (ALS/PDC) observed among the native Chamorro people. This hypothesis was later criticized and rejected [3] and subsequently reinforced in the early 2000s. At this time, a group demonstrated successively that BMAA can be produced by a symbiotic cyanobacteria (*Nostoc* sp.) [4], that BMAA can be biomagnified within the Guam ecosystem, from cyanobacteria to the brains of people who died from ALS/PDC [4,5,6], and that large amounts of BMAA can be released from proteins after total acid hydrolysis of samples [5].

After Cox *et al.* [7] reported that almost all cyanobacteria can produce BMAA, other groups have found BMAA in different ecosystems all around the world [8,9,10,11,12], and three patterns of biomagnification were also suggested, respectively, in the Baltic Sea [13], in the Florida Bay [14] and in Lake Taihu, China [15]. Taken together with the observation of BMAA in the brains of Canadian patients who suffered from several neurodegenerative diseases [16], these findings suggested a possible widespread human exposure to the BMAA neurotoxin and its global implication in ALS and also in Alzheimer’s disease and Parkinson’s disease.

However, the presence of BMAA in cyanobacteria and other matrices is controversial [17,18]. Indeed, the classically-used fluorescent mode of detection after derivatization of samples is known to lack selectivity [19], and high amounts of BMAA were reported with this method (up to 7000 µg/g dry weight). On the opposite side, when more selective methods, like LC-MS/MS, were employed, concentrations or even the presence of BMAA were not confirmed [12,19,20,21,22]. The derivatization allows for an increase of the molecular weight of molecules, thus reducing the background signal while improving the ionization efficiency with electrospray ionization systems [23]. Nevertheless, the derivatization is not specific; it affects the chromatographic separation and leads to the indirect detection of BMAA [17]. Discrepancies observed among the BMAA concentrations reported likely originate from the lack of selectivity of the methods employed and not because of the use of a derivatization step [18]. Indeed, highly selective methods have been validated for BMAA and analogues [24,25].

The existence of at least three natural isomers, 2,4-diaminobutyric acid (DAB), *N*-2-aminoethylglycine (AEG) and β-amino-*N*-methyl-alanine (BAMA), can be partly involved in BMAA controversy and highlighted the requirement of highly selective methods to unambiguously detect and quantify BMAA from its isomers. DAB is a neurotoxic isomer of BMAA [17] that was first found in cyanobacteria in 2008 [22], but it has also been widely reported in prokaryotes and eukaryotes [23]. Among all known isomers of BMAA, AEG and BAMA were selected by two groups for method development, because they could potentially interfere with BMAA analysis [26,27]. Indeed, AEG was found in cyanobacteria, and its production was suggested to be highly conserved [28], while BAMA was observed in mollusks of the Baltic Sea [27]. The toxicity of these two isomers has not been studied so far, especially for AEG.

To date, two papers have reported BMAA in cyanobacteria with selective MS/MS methods using a derivatization of samples [25,29]. Recently, BMAA was also reported to be produced by diatoms and dinoflagellates [30,31,32]. However, the free form of BMAA was not considered. It is unclear why the free form of BMAA is no longer analyzed, since it seems reasonable to assume that biomagnification could more easily originate from the free rather than the bound form of BMAA. Indeed, Dunlop *et al.* [33] have reported that free BMAA can be misincorporated into human neuroproteins instead of serine in an *in vitro* cell line, and this incorporation into proteins had been initially postulated as the mechanism of the bioaccumulation of BMAA in the Guam ecosystem [5].

BMAA is a small molecule that can be directly detected by mass spectrometry (MS), but the use of a derivatization step is also chosen by some groups, followed by either fluorescent (FLD) or MS detection. To cope with the complexity of biological matrices and the controversy arising from detecting trace amounts of BMAA, we aimed at the development of a highly selective and sensitive LC-MS/MS method to confidently quantify both free and bound forms of BMAA, DAB and AEG. Thus, following the recommendations of Cohen [34], we included a solid phase extraction (SPE) clean-up step in the sample preparation procedure and the use of an isotopically labeled molecule (*i.e.*, deuterated D_5_DAB) as the internal standard. There are ongoing debates about the use of a derivatization step [17,20,23]. While the derivatization approach is interesting in combination with mass spectrometry, there is little information in the literature on the derivatization yield for BMAA [34]. A lot of compounds can react with the 6-aminoquinolyl-*N*-hydroxysuccinimidyl carbamate (AQC) reagent during the derivatization process [17]. Furthermore, the analysis of BMAA and analogues is already rather complex, and any additional step could contribute to recovery losses and add variability to the method. Additionally, the chromatographic separation of the derivatized analogues is poor compared to that of the underivatized compounds [17]. We therefore chose not to use a derivatization step in our analytical approach. We decided to optimize the analysis of underivatized BMAA and isomers (DAB and AEG) with HILIC chromatography coupled to a sensitive triple quadrupole mass spectrometer (QTRAP 5500 system). This optimized procedure was then applied to screen ten species of cyanobacteria and nineteen samples of mollusks collected in 2009 in Thau Lagoon, French Mediterranean, where BMAA and DAB were recently reported in mussels and oysters with an LC-MS/MS method employing derivatization of the samples [35].

## 2. Results and Discussion

### 2.1. Optimization of the Analysis of BMAA and Isomers

The analysis of BMAA requires several steps. After performing cell lysis in an adapted solvent, samples should be cleaned up using an SPE procedure to remove interfering compounds and then injected into an LC-MS/MS system to take advantage of the higher selectivity of this hyphenated technique. BMAA can be extracted either as the free or as the bound form. To release the BMAA bound to proteins, an acid hydrolysis step has to be added before SPE clean-up.

#### 2.1.1. Cell Lysis and Solvent Extraction

To extract cyanotoxins, including BMAA (both in free and protein-bound form), cells have to be disrupted in a solvent facilitating the solubilization of the analytes. Generally, freeze-thawing and maceration [36] or sonication [37] in an appropriate solvent were employed. However, the cell lysis of cyanobacteria can be hard to achieve [38]. Using a mixer mill, excellent disruption of cyanobacteria was observed (checked by microscopic observation). The protocol, proposed by Serive *et al.* [39], was adapted to extract polar and basic compounds from freeze-dried material.

**Figure 1:**
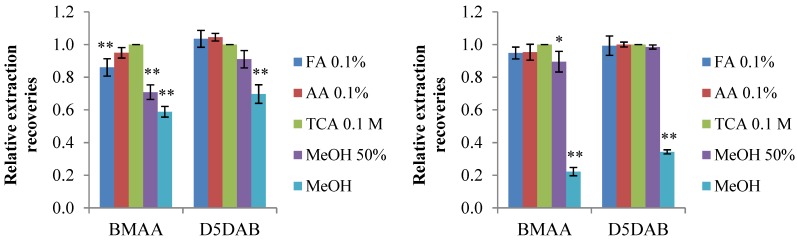
Extraction recoveries of BMAA and D_5_DAB spiked at 30 ng/mL in (**A**) cyanobacteria and (**B**) mussel matrix with five different solvents (*n* = 3, error bars represent standard deviations). Results are expressed relatively to the recoveries in trichloroacetic acid (TCA) 0.1 M. * *p* < 0.01 and ** *p* < 0.001 compared to TCA 0.1 M. FA, formic acid; AA, acetic acid.

To select an adapted solvent of extraction, both freeze-dried cyanobacteria *Leptolyngbya* PCC 73110 and mussel (*Mytilus galloprovincialis*) were spiked in triplicate with BMAA and D_5_DAB before grinding and extracted with five solvents. To date, different solvents have been used to extract free, bound or total BMAA [34]. We compared the recoveries for free BMAA and its isomers between formic acid (FA) 0.1%, acetic acid (AA) 0.1%, trichloroacetic acid (TCA) 0.1 M, methanol (MeOH) and MeOH/water-FA 0.1% (50:50, v/v). The solvents were chosen accordingly to the literature: e.g., TCA 0.1 M is classically used to extract BMAA [34]; acetic acid is employed to extract paralytic shellfish poisoning (PSP) toxins (basic polar compounds) [40]; while MeOH is known to be able to extract marine toxins of a wide range of lipophilicity from wet algae [41]. The samples were analyzed after SPE clean-up as detailed in the Experimental Section (Section 3.3.2). The results obtained were expressed relatively to the recoveries in the TCA 0.1 M for cyanobacteria and mussel matrices (Figure 1), because it is widely used to extract free BMAA.

**Figure 2:**
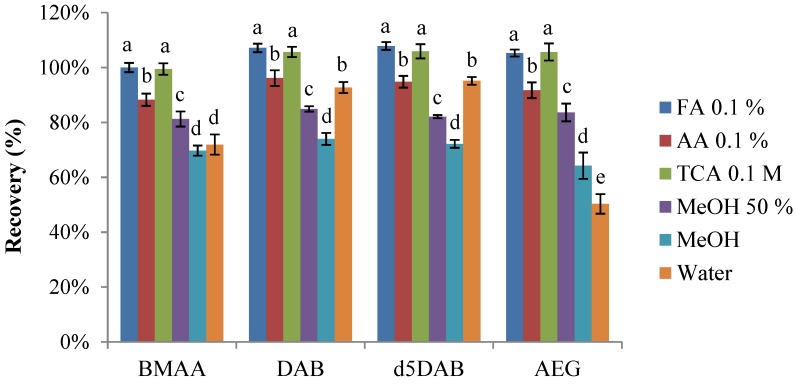
Recoveries of pure standards spiked at 30 ng/mL in different extraction solvents (*n* = 3, error bars represent standard deviations). Replicates with the same letter are not significantly different, while replicates with different letters are significantly different (*p* < 0.05).

For D_5_DAB, all solvents, but pure MeOH (*p* < 0.001), gave similar recoveries. For BMAA, the performances of acidic solvents are alike (except FA 0.1% with cyanobacteria), but lower recoveries were observed in the presence of methanol. The poor recoveries obtained with pure methanol for both matrices were expected, as BMAA and D_5_DAB are polar compounds. Indeed, the recoveries of pure standards were only about 70% (Figure 2) when using methanol as the extraction solvent, which may indicate low solubility and/or adsorption to glass vials, as suggested by Cohen [34]. Considering the recoveries of standards (Figure 2) and the recoveries obtained with spiked matrices (Figure 1), we chose TCA 0.1 M as the solvent for the extraction of free BMAA and isomers.

Subsequently, the protocol was adapted to extract total rather than the bound form of analytes, since the precipitation of proteins is time-consuming and difficult [34]. The classical acid hydrolysis in boiling hydrochloric acid (HCl) 6 M [5] was performed following extraction with TCA 0.1 M.

#### 2.1.2. Comparison of Solid Phase Extraction (SPE) Sorbents

**Figure 3:**
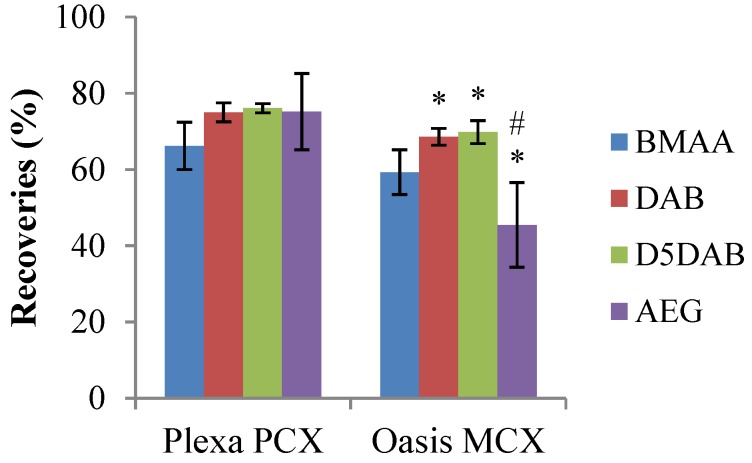
Recoveries of mixed solutions of standards spiked in TCA 0.1 M at a final concentration of 30 ng/mL. Comparison between 60 mg/3cc Plexa PCX and Oasis^®^ MCX cartridges (*n* = 3, error bars represent standard deviations). * *p* < 0.05 compared to recoveries with Plexa PCX. ^#^ *p* < 0.005 compared to D_5_DAB and DAB with Oasis^®^ MCX.

The SPE clean-up step is necessary to remove co-eluting interfering compounds that cause matrix effects during LC-MS/MS analysis while concentrating samples [42,43,44,45]. As basic amino acids, BMAA and isomers (DAB, D_5_DAB and AEG) can be purified with cation-exchange sorbents, such as Strata SCX (Strong cation exchange) [46], or mixed-mode sorbents, like Oasis^®^ MCX [21] or Isolute^®^ HCX-3 [25]. Here, the widely-used Oasis^®^ MCX [21,24,45] was compared to its Agilent equivalent, Bond Elut^®^ Plexa PCX sorbent (Agilent Technologies, Les Ulis, France), for the recovery of the standards (Figure 3).

Recoveries with Plexa PCX were better than those obtained with Oasis^®^ MCX cartridges (*p* < 0.05), except for BMAA. The recoveries obtained here with Oasis^®^ MCX were lower than those obtained for BMAA and DAB by other groups [24,45], possibly related to small differences in protocols. As extraction was performed in TCA 0.1 M, we decided to directly percolate samples in TCA after activation of the cartridges (2 mL of MeOH, then 1 mL of TCA 0.1 M). Indeed, due to their pKa (>6.5) [45], the pH of TCA 0.1 M allows for the ionization of ammonia groups and, hence, retention of positively-charged molecules on the sorbent. To increase recoveries (especially for AEG; data not shown), we used an elution solvent consisting of a mixture of MeOH/NH_4_OH (93:7 instead of 95:5, v/v). Finally, as trace levels of BMAA and isomers (about 15% of the concentration of the spiking solution) were found in fractions eluting after the 3 mL used by Combes *et al.* [24], an additional 1 mL was used for the elution step.

It has to be noted that an unknown peak eluted close to the AEG retention time in blank (1 mL TCA 0.1 M) passed through both types of cartridges during LC-MS/MS analysis. However, this unknown compound originating from sorbents is different from the one reported by Li *et al.* with the Strata-X-C cartridge [45], since only the transition *m/z* 119 > 102 gave a signal. To accurately quantify AEG, the peak area of this interfering compound was subtracted from the peak area of AEG in samples.

The SPE clean-up procedure with PCX cartridges, as described in the Experimental Section (Section 3.2.2), was applied to all samples after extraction of both free and total BMAA and its isomers.

#### 2.1.3. Optimization of Selectivity: Chromatographic Resolution and Mass Spectral Transitions

Among cyanobacterial toxins, BMAA is a compound that has caused controversy, due to the confusion arising from interferences in the analytical determination [18]. Indeed, at least three isomers of BMAA—DAB, AEG and BAMA—can be found in biological matrices [12,25,30,32]. The large discrepancies of BMAA concentrations observed between studies were suggested to come mainly from the lack of selectivity of the methods that have been used [18]. Therefore, we decided to maximize both chromatographic resolution and the selectivity of mass spectral transitions during the method development.

**Figure 4:**
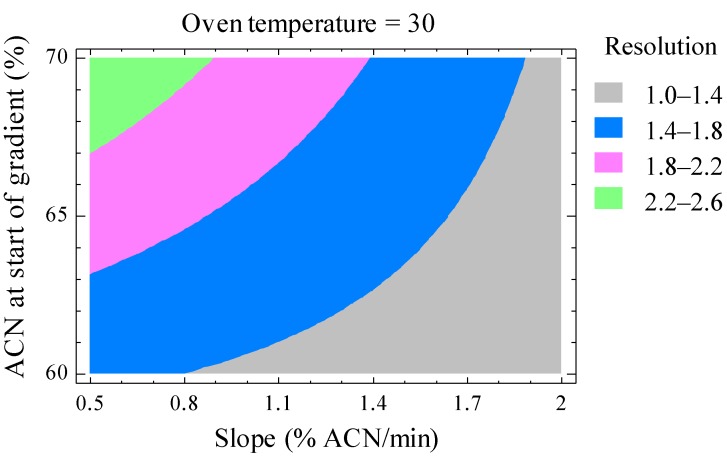
Chromatographic resolution between BMAA/DAB, optimized using a 2^3^ factorial design. Since temperature did not influence the resolution significantly, it was set to 30 °C. A resolution of 1.5 corresponds approximately to the baseline resolution. ACN, acetonitrile.

To have a proper identification of BMAA and isomers, chromatographic resolution has to be reached, since the common mass spectral transition *m/z* 119 > 102 is used to quantify underivatized samples. For this purpose, a gradient elution with a ZIC^®^-HILIC column was optimized using a 2^3^ factorial design. The main effect and interaction of three variables, namely acetonitrile (ACN) at the start of gradient (60%–70%), oven temperature (25–35 °C) and the slope of the gradient (0.8%–1.5% ACN/min), were studied on BMAA/DAB and DAB/AEG resolutions. Injections were made twice, which led to 16 experiments, and then, a response surface was generated (Figure 4).

**Figure 5:**
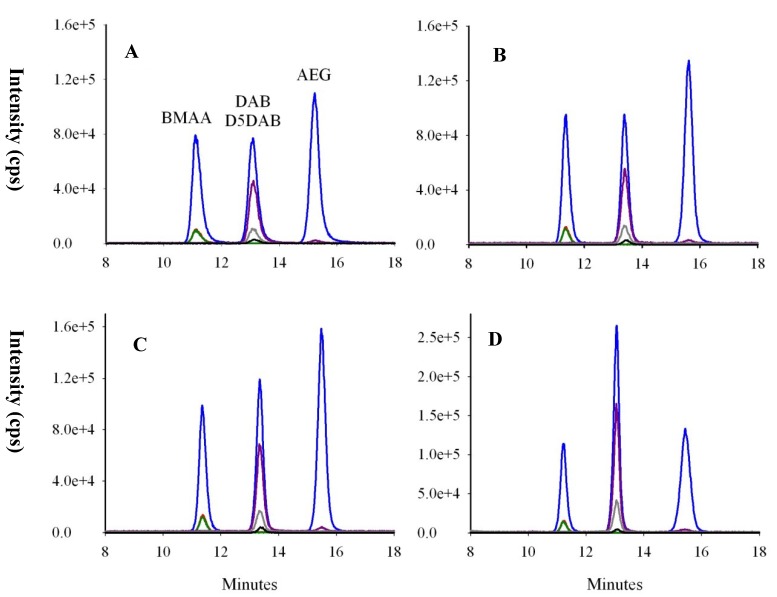
Chromatograms of BMAA, D_5_DAB and AEG spiked at 50 ng/mL into (**A**) standards; (**B**) cyanobacteria; (**C**) oyster and (**D**) mussel matrices after extraction of free analytes. Colored lines represent mass spectral transitions at *m/z* 119 to *m/z* 102 (blue), 88 (red), 76 (green), 101 (purple), 74 (grey) and *m/z* 124 to *m/z* 47 (dark).

Hydrophilic interaction liquid chromatography (HILIC) columns are designed to efficiently separate polar compounds [47]. The ZIC^®^-HILIC column has been successfully used to separate BMAA and DAB [11,19,22,24], and the response surface obtained after the factorial design here clearly shows that excellent resolutions can be achieved with this column for underivatized molecules (BMAA, DAB and also AEG). Conditions were set to have a baseline resolution of 1.5 between molecules, as can be seen in Figure 5. Peak maxima were *ca*. 2 min apart, with a baseline separation of 1 min typically being achieved, thus reducing the risk of misidentification of BMAA, DAB and AEG, while limiting the co-elution of interfering compounds causing matrix effects.

Classically, LC-MS/MS analysis is preferred to quantify BMAA, because of the four criteria for the identification of molecules (retention times, parent ion, product ion(s) and ion ratios) [18]. For maximum selectivity, we decided to consider not only one (as methods involving derivatization do [25]), but two specific (qualitative) transitions for BMAA and DAB and the ratios between qualitative and quantitative ions (respectively, *m/z* 119 to *m/z* 88 and 76 for BMAA and *m/z* 119 to *m/z* 101 and 74 for DAB; ratios 88/102, 76/102, 101/102 and 74/102). For AEG, no specific mass spectral transition was found (*m/z* 119 > 41 was specific, but suffered from a high background signal). Generally, the ratios of the qualifier ion(s) to the quantifier ion that are used to confirm the identity of molecules in samples should be within a 10% error range of the ratios of standards [27], which was the case in our study (Table 2). Finally, we have developed a highly selective method to confidently identify BMAA and isomers in biological samples.

### 2.2. Characterization of the Analytical Procedure

#### 2.2.1. Recoveries of SPE Clean-Up and Remaining Matrix Effects

SPE recoveries and matrix effects were assumed to have a significant impact on the analysis of BMAA and isomers. We aimed to screen cyanobacteria, but also mollusks, for the presence of BMAA, DAB and AEG. After the selection of the Plexa PCX cartridge, recoveries of the SPE clean-up with representative matrices (cyanobacteria, oyster and mussel) and the remaining matrix effects were assessed for extraction procedures of both free and total BMAA and isomers (Table 1).

**Table 1:** Recoveries (*n* = 3) of the solid phase extraction (SPE) clean-up (spiked solutions at 30 ng/mL) and remaining matrix suppression of the three matrices after the extraction of free and total BMAA and isomers. Matrix effects were evaluated as explained in Section 3.5.

Extraction	Matrix	Recoveries of SPE Clean-Up (mean% ± SD)				Matrix Effect (%)			
		BMAA	DAB	D5DAB	AEG	BMAA	DAB	D_5_DAB	AEG
Free analytes	Cyanobacteria	61.1 ± 4.9	51.0 ± 5.6	61.0 ± 1.6	48.1 ± 5.5	8.8	5.6	4.7	9.3
	Oyster	56.1 ± 4.5	64.5 ± 4.5	65.4 ± 4.5	63.1 ± 3.8	7.3	0.9	−3.5	0.2
	Mussel	63.8 ± 3.1	79.0 ± 6.8	75.6 ± 5.7	73.3 ± 2.1	5.3	−6.3	1.2	0.7
Total analytes	Cyanobacteria	67.6 ± 3.3	63.1 ± 1.9	63.4 ± 7.5	59.5 ± 1.0	3.8	1.9	4.2	−9
	Oyster	71.0 ± 2.0	76.4 ± 9.5	81.1 ± 4.1	74.0 ± 6.4	7.5	3.5	3	3.6
	Mussel	64.3 ± 8.0	65.0 ± 8.1	73.1 ± 6.5	64.3 ± 0.9	8.7	12.1	15.7	−5.5

Bond Elut^®^ Plexa PCX is a mixed-mode SPE sorbent, which contains a highly polar polymeric cation-exchange resin with strong cation exchange functionalities. According to the manufacturer [48], Plexa PCX removes neutral and acidic interferences from the matrix and concentrates basic analytes, like BMAA and isomers. Ion suppression is reduced, because the highly polar, hydroxylated polymer surface is entirely amide-free and does not provide binding sites for endogenous species, such as proteins and lipids.

Recoveries of BMAA and isomers are globally within the same range (≥60%). Surprisingly, recoveries of analytes from cyanobacterial matrix were on average lower than those from the two more complex mollusk matrices. The cation-exchange functionality did not suffer from the higher content of amino acids in samples after acid hydrolysis, since similar recoveries were observed for the extraction of both free and total amino acids. A decrease of SPE recoveries due to the presence of matrix components can be seen in comparison with recoveries obtained with standards, as shown by Kasprzyk-Hordern [49]. Recoveries obtained for BMAA and DAB are comparable to those reported by other groups using Oasis^®^ MCX cartridges and HILIC-MS methods to analyze cyanobacteria (>70% for Li *et al*. [45] and Combes *et al.* [24], but >80% for Kubo *et al.* [21]). Overall, the recoveries of the methods reported in the literature for the quantification of BMAA in cyanobacteria are generally better without SPE clean-up; however, matrix effects have not always been evaluated [19,20]. We could not find any information on SPE recoveries of underivatized analytes in mollusk matrices.

The matrix effects of BMAA and especially its isomers are not well documented. Li *et al.* [45] have reported no effect on BMAA, but DAB suppression with hydrolyzed cyanobacterial matrix, while strong matrix effects (>30%) were still present after SPE clean-up of cyanobacteria and biofilms in the procedure of Combes *et al.* [24]. Due to previous studies in our laboratory [50], matrix effects caused by mollusks were assumed to be higher than those caused by microalgae. However, this was not the case for this analyte-matrix combination.

**Figure 6:**
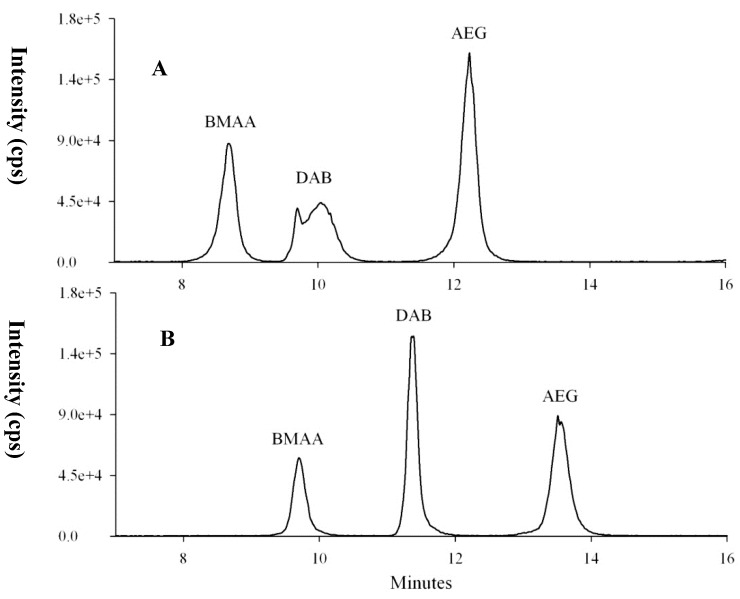
The effect of the SPE clean-up on mussel matrix spiked with BMAA and AEG after extraction of free amino acids (DAB naturally present in the sample). The black line corresponds to the mass spectral transition *m/z* 119 > 102. (**A**) Spiked mussel extracted with TCA 0.1 M and injected in this solvent, while (**B**) the same sample subjected to PCX SPE clean-up and injected in ACN/water/FA. The shift in retention times compared to other chromatograms is due to the non-use of the guard column in (B) and due to the injection solvent, matrix effects and the non-use of a guard column in (A).

After our SPE clean-up protocol, the remaining matrix effects, *i.e.*, signals suppression or enhancement in the electrospray interface of LC-MS/MS, were relatively low for the three underivatized matrices (less than 15% of loss after extraction of both free and total amino acids). This suggests that our method was not affected by the strong signal suppression due to BMAA reactivity (*i.e.*, metal adduct formation), as recently reported by Glover *et al.* [51] for the analysis of underivatized BMAA. Despite somewhat lower recoveries than reported elsewhere, the proposed SPE protocol seems to be very effective at cleaning cyanobacteria and mollusk matrices, while reproducibly extracting BMAA and isomers. For example, a strong matrix effect was observed for DAB in mussel matrix without the SPE clean-up step, while peak shapes and retention times were better and closer to the standards after SPE clean-up (Figure 6).

Among the internal standards that have been used, deuterated-BMAA, *i.e.*, D_3_BMAA [22,25], and ^5+^BMAA [52] are more pertinent to accurately quantify samples by applying a corrective factor. In the absence of commercially available deuterated-BMAA (or otherwise isotopically-labeled BMAA), a group has recently validated a procedure to quantify underivatized BMAA and DAB using D_3_DAB as the internal standard [24]. As a result, we chose D-2,4-diaminobutyric acid-2,3,3,4,4-^2^D_5_ dihydrochloride (D_5_DAB) as the internal standard. D_5_DAB was expected to have recoveries and matrix effects similar to DAB and presumably similar to BMAA and AEG. This hypothesis was confirmed since concentrations obtained after correction with D_5_DAB recovery are within a 15% error range (20% for free AEG in cyanobacteria) and not overestimated. Therefore, a corrective factor based on D_5_DAB recovery could be applied to more accurately quantify BMAA and isomers in cyanobacteria, oyster and mussel matrices.

#### 2.2.2. LC-MS/MS Performance: Linearity, LOD, LOQ and Repeatabilites (RT, *R*^2^ and Ion Ratios)

The calibration curves for BMAA, DAB and AEG showed good linearity (*R*^2^ > 0.9996) over the concentration range 1–500 ng/mL. However, a five-point calibration curve ranging from 1 to 100 ng/mL was classically used to quantify samples. The equations for the linear regressions were: *y* = 42,414*x* − 6703 for BMAA, *y* = 41,400*x* − 15,604 for DAB, *y* = 66,882*x* − 44,981 for AEG and *y* = 1252*x* − 585 for D_5_DAB.

The limit of detection (LOD) was defined as the lowest concentration giving a signal-to-noise (S/N) ratio of three (standard deviation of noise = σ; S/N = 3 σ) for the four qualitative mass spectral transitions of BMAA and DAB. The limit of quantification (LOQ) was calculated using the general transition *m/z* 119 > 102. Because the area of the peak at *m/z* 102 was more than seven-times higher than the peaks at *m/z* 76 and 74, the LOQ (with S/N > 10, σ = 3) was defined as equal to the LOD.

The LOQ was 3 ng/mL for standards and equivalent to 15 pg on the column. The LOQs within matrices were calculated by spiking freeze-dried material subjected to the extraction of both free and total analytes, and they were 0.225 µg/g dry weight (DW) of cyanobacteria and 0.15 µg/g DW of shellfish. However, since no clean mollusk matrices were found (especially for DAB and BMAA after extraction of total amino acids), LOQs for mollusks were estimated based on matrix effects. The difference of LOQs observed between cyanobacteria and mollusk matrices relied on the amount of freeze-dried material extracted (respectively, 10 and 15 mg).

While having an optimized selectivity, the instrument and method sensitivity are among the best reported for HILIC methods [19,20,22,24,45], but inferior to those reported for derivatized analytes in cyanobacterial samples [25,35]. The LOQ we obtained for cyanobacteria was better than the LOQ reported in the procedure of Combes *et al.* [24], possibly due to higher matrix effects in their HILIC-MS/MS method. For mollusk matrices, no LOQ of underivatized BMAA and isomers is reported. Nevertheless, LOQs of 1.7 µg/g DW and 0.15 µg/g wet weight were reported for derivatized BMAA with a gastropod [53] and a mussel matrix [54].

Thanks to an effective clean-up step and optimized gradient elution, we obtained a sensitive and selective method to quantify BMAA, DAB and AEG in cyanobacteria and mollusk matrices.

The repeatability of retention times, the correlation coefficient (*R*^2^) and ion ratios of standards calibration curves (*n* = 33) and spiked matrices are shown in Table 2.

Even if no clean matrices were found to assess the matrix effects, the strategy adopted (see Section 3.5) allowed for the good linearity of spiked matrices. Indeed, *R*^2^ > 0.998 with extraction of free amino acids and *R*^2^ > 0.99 (with some exceptions) after extraction of total amino acids were observed. The lower (*R*^2^ ≤ 0.98) coefficient of determination for DAB (oyster and mussel) came from the natural high content of this isomer in the mollusk matrices used.

Despite the presence of a relatively low matrix effect, the retention times of analytes and the ion ratios were rather stable. The good repeatability obtained (*i.e.*, relative standard deviations less than 1% and 10% for RT and ion ratios, respectively) made this HILIC-MS/MS method suitable for the accurate quantification of BMAA and isomers in cyanobacteria and mollusk matrices, after extraction of both free and bound BMAA and its isomers.

**Table 2:** Retention times, correlation coefficients (*R*^2^) and ion ratios of standards’ curves and spiked cyanobacteria (*Leptolyngbya* PCC73110), oyster and mussel matrices (mean ± SD) for the extraction of free and total amino acids. For standards, the results came from 33 injections of the five-point calibration curves. For matrices, results originate from the established three-point standards’ curves (3–50 ng/mL) for matrix effects injected twice (for *r*^2^, *n* = 2) and both matrix effects and SPE recoveries for RT and ion ratios (*n* = 6).

Samples	RT (min)				*R*^2^				Ion Ratios (%)			
	BMAA	DAB	D_5_DAB	AEG	BMAA	DAB	D_5_DAB	AEG	88/102	76/102	101/102	74/102
**Standards**	11.25 ± 0.08	13.27 ± 0.10	13.34 ± 0.11	15.47 ± 0.11	0.9999	0.9998	0.9996	0.9996	13.1 ± 0.8	12 ± 0.8	60.3 ± 2	14.2 ± 0.8
**73110 ^a^**	11.42 ± 0.05	13.39 ± 0.08	13.47 ± 0.08	15.50 ± 0.13	0.9997	0.9995	0.9995	0.9995	13.0 ± 0.1	12.0 ± 0.4	58.8 ± 2	14.7 ± 1
**73110 ^b^**	11.12 ± 0.04	13.03 ± 0.04	13.08 ± 0.04	15.17 ± 0.09	0.9998	0.9973	0.9992	0.9990	12.6 ± 1	11.5 ± 1	58.9 ± 4	14.3 ± 1
**Oyster^ a^**	11.42 ± 00.5	13.41 ± 0.06	13.44 ± 0.07	15.55 ± 0.06	0.9999	0.9991	0.9997	0.9993	12.4 ± 1	11.3 ± 1	58.6 ± 1	14 ± 0.4
**Oyster ^b^**	11.06 ± 0.04	12.82 ± 0.09	12.88 ± 0.10	15.08 ± 0.14	0.9986	0.9807	0.9993	0.9915	12.0 ± 0.5	10.5 ± 0.4	58.4 ± 2	14.4 ± 0.6
**Mussel ^a^**	11.30 ± 0.07	13.14 ± 0.07	13.17 ± 0.07	15.46 ± 0.11	0.9997	0.9983	0.9996	0.9995	12.8 ± 0.9	11.5 ± 0.9	59.4 ± 0.6	14.6 ± 0.3
**Mussel ^b^**	10.91 ± 0.03	12.53 ± 0.05	12.53 ± 0.05	15.14 ± 0.11	0.9975	0.9498	0.9990	0.9919	12.0 ± 0.3	10.5 ± 0.4	58.5 ± 0.7	14.4 ± 0.4

RT, retention time; ^a^ samples spiked after extraction of free amino acids; ^b^ samples spiked after extraction of total amino acids.

### 2.3. Screening of BMAA and Isomers in Cyanobacteria

#### 2.3.1. Kinetics of Growth and Production of BMAA and Isomers

**Figure 7:**
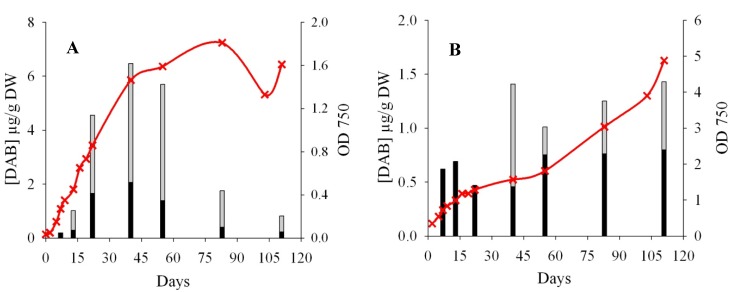
Growth curves (red line, cross symbols) and free (dark histograms) and bound (grey histograms) DAB concentration for (**A**) *Nostoc* CCMP 2511 and (**B**) *Leptolyngbya* PCC 73110.

As the production of BMAA by cyanobacteria has been suggested to be “a function of growth condition and/or life cycle stages” [7], the kinetics of growth and toxin production in batch cultures were made with two non-axenic strains that had previously been reported to produce BMAA: *Leptolyngbya* PCC 73110 [12,25] and *Nostoc* CCMP 2511 [36]. The *Nostoc* strain was reported to produce BMAA by methods lacking in selectivity (e.g., FLD), while *Leptolyngbya* was recently quantified with a highly selective MS/MS method. Biomass was regularly monitored, and cells were harvested between seven to 111 days of growth. The extraction of free analytes was performed on all samples, while the extraction of free plus bound (=total) analytes was carried out when freeze-dried material was available (Day 13 for both *Nostoc* CCMP 2511 and Day 40 for *Leptolyngbya* PCC73110 (Figure 7).

Neither free nor bound BMAA were found in any of the two strains, at any time of the growth curve. However, these strains are known to be BMAA producers, and the reported concentrations are above our LOQ [25,36]. For the *Nostoc* strain, the use of a non-selective method could have led to misidentification and/or overestimation of BMAA in previous studies [18]. Nevertheless, Jiang *et al.* have reported 0.73 µg/g DW of BMAA in the *Leptolyngbya* PCC73110 thanks to a highly selective and validated MS/MS method involving the derivatization of samples [25]. The conditions of the culture that we and their group applied are really close [12], and the selectivity of both methods drastically reduces the risk of misidentification. It should be noted that this group had performed a total extraction of BMAA after six days of growth, while we could only perform total extraction after 40 days of growth (due to limited freeze-dried material).

The fact that “cyanobacteria in culture display a fluctuating BMAA production”, as suggested by this same group [30], may explain why we could not find BMAA in our *Leptolyngbya* culture, if the production is highly variable. Further trials were made with this strain using different sources of nitrogen in the culture medium and different culture durations; however, BMAA was never found in our work, while AEG was sometimes detected (data not shown).

Interestingly DAB, a possible neurotoxic isomer of BMAA [23], was detected in all samples analyzed. As observed for BMAA [7], no correlation between the free and bound form of DAB was observed, but the bound form seemed to be more important. For example, free DAB from LOQ to 2 µg/g was reported for CCMP 2511, while the total form ranged from 0.82 to 5.8 µg/g DW. The kinetic of DAB production (both free and bound) by CCMP 2511 seemed to follow the kinetics of growth (Figure 7A). On the contrary, DAB production by the other strain seemed more stable and independent of growth. The two kinetics of DAB production were different, and the concentrations found varied by up to one order of magnitude; however, DAB was always detected in these two strains during the kinetic experiment.

#### 2.3.2. Screening of Others Lab-Cultured Cyanobacteria

We screened eight other cyanobacterial strains for the production of BMAA and isomers (Table 3). Unlike Banack *et al.* [28], we could not find AEG in *Synechocystis* sp. PCC 6803 and *Nostoc* PCC 7120 extracts, but trace levels (from LOD to 1.5 µg/g DW of total AEG) were sometimes found in *Leptolyngbya* PCC 73110, *Synechococcus elongatus* CCAP1479/1B and *Nostoc endophytum* CCAP1453/14 (data not shown).

Besides the three strains above, four more cyanobacteria were screened, as they had been previously reported to produce BMAA by the non-highly selective FLD [7] or selective MS/MS method with derivatization [29]. Finally, more than 60 samples corresponding to ten strains were analyzed with the HILIC-MS/MS method that we have optimized. No BMAA was found, while at least trace amounts of DAB were observed in all samples (except for PCC6803). The lack of selectivity of FLD is now well admitted, so the high concentrations of BMAA [7] reported with this detection technique cannot be fully considered. However, DAB production by cyanobacteria had already been reported, in *Calothrix* (<50 µg/g) in 2008 [22], then in 16 cyanobacterial samples (<0.83 µg/g) in 2010 [20] and in trace amounts in 2012 [45].

**Table 3:** The content of free and total DAB (µg/g DW) in ten strains of cyanobacteria. When more than one discrete sample was screened (different medium of culture or days of growth), the minimum and the maximum concentrations are reported.

Cyanobacterial Strain	Free DAB (µg/g DW)	Total DAB (µg/g DW)
*Leptolyngbya* PCC 73110 ^a^	<LOD–2.69	1.01–1.57
*Nostoc* CCMP 2511/ CMMED01 ^a^	<LOD–2.27	0.82–12.48
*Microcystis* PCC 7806 ^a^	<LOD	<LOD
*Nostoc* PCC 7120 ^a^	<LOD	*
*Nostoc* PCC 7107 ^a^	2.12–7.2	*
*Symploca* PCC 8002 ^a^	0.3	0.43
*Synechocystis* sp.PCC 6803	<LOD	<LOD
*Synechococcus elongatus* CCAP1479/1B	0.23–2.71	0.4–3.52
*Calothrix crustacea* CCAP1410/9	0.6–0.92	6.95–14.53
*Nostoc endophytum* CCAP1453/14	0.34–0.55	6.78–7.52

* Not analyzed for total DAB; <LOD, a peak corresponding to the transition 119 > 102 was detected at a retention time close to D_5_DAB, but was not quantifiable (<0.225 µg/g); ^a^ strain reported to be a BMAA producer.

In these cases, HILIC methods were used, but DAB was also observed after derivatization of *Leptolyngbya* PCC73110 [12]. Once again, the bound form of DAB seemed to predominate. The concentrations we obtained here for DAB varied by a factor of 10 to 15 within the same strain, yet they still were within the concentration ranges reported before.

Krüger *et al.* [17] have raised a possible substance conversion into DAB during the hydrolysis of the sample preparation procedure, but we did detect free DAB with our procedure to extract free amino acids in which no acid hydrolysis of the samples was performed.

Little is known about BMAA and DAB production by cyanobacteria. The only work focusing on the factors that influence BMAA production showed that nitrogen starvation resulted in the production of BMAA [55]. However, the methodological approach and conclusion of this study were subsequently criticized [18]. This same group has recently reaffirmed a link between the content of BMAA and nitrogen availability in a field study of cyanobacterial blooms [56]. They have also suggested that “at high nitrogen level (>40 µM), BMAA production is suppressed” [56]. However, this is not confirmed by the publication of Jiang *et al.* [25] in which BMAA was reported in lab-cultured *Leptolyngbya* PCC 73110 grown in BG11 (Blue-Green 11) medium that contains a very high nitrate concentration. We also could not confirm this hypothesis with our lab-cultured cyanobacteria. Indeed, we grew cultures of *Nostoc* CCMP 2511 and *Leptolyngbya* PCC 73110 in media without any nitrogen source and did not observe the appearance of BMAA production in those strains that had been reported to produce BMAA before.

Now, highly selective methods exist, with and without a derivatization step [24,25], so the ability of cyanobacteria to produce BMAA and the factors controlling this production can be effectively assessed. In this attempt, only two strains (*i.e.*, *Leptolyngbya* PCC73110 and *Nostoc* PCC7120) were confirmed to contain BMAA after derivatization of samples [25,29]. However, we could not find BMAA in these two strains with our selective HILIC-MS/MS method, maybe due to different conditions of culture [45], or because of the supposed brief nature of BMAA production by cyanobacteria [30], or due to the lack of sensitivity of our method. Nevertheless, we did detect DAB in almost all samples and sometimes AEG, which stresses the requirement of highly selective methods to accurately quantify BMAA in cyanobacteria.

#### 2.3.3. Screening of Mollusks of Thau Lagoon

Since the discovery of BMAA in 1967, the link between BMAA and neurodegenerative diseases, like amyotrophic lateral sclerosis (ALS), is still under debate [3,57,58]. The consumption of contaminated aquatic organisms/seafood is a possible pathway of human exposure to BMAA. Potential associations of BMAA found in aquatic organisms with sporadic ALS were recently hypothesized in Chesapeake Bay, Maryland, USA [59], and in Thau Lagoon, France [35], with methods using the derivatization of analytes.

We decided to screen BMAA and isomers in mollusks collected in the Thau Lagoon during summer, 2009, with the HILIC-MS/MS that we have developed.

**Figure 8:**
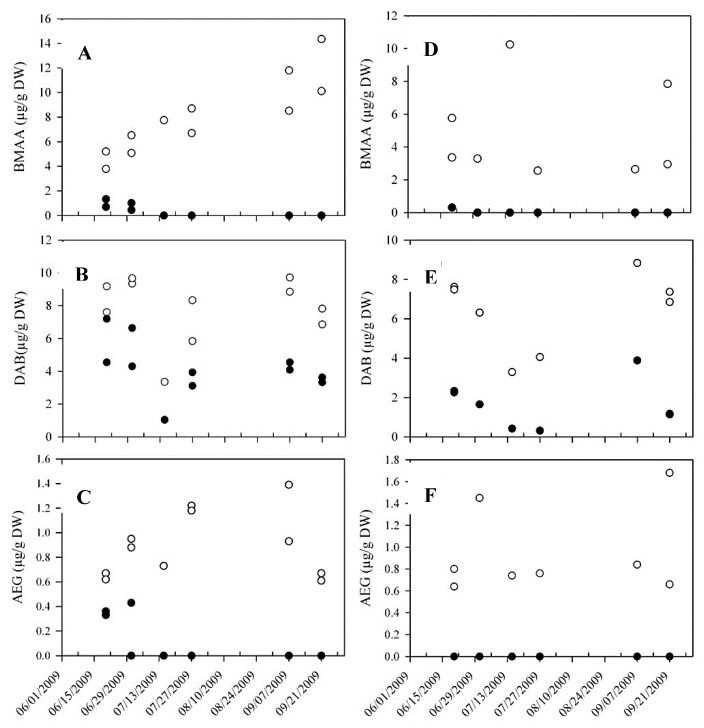
(**A**–**C**) BMAA, DAB and AEG concentrations in free (black circles) and total form (empty circles) in mussels collected in 2009 and (**D**–**F**) in oysters. Mollusks were sampled in two of the three areas where shellfish were cultured in the lagoon and analyzed for both areas when material was available.

The three isomers, BMAA, DAB and AEG, were found in mussels and oysters of this bivalve farming area (Figure 8). The samples were freeze-dried and stored at room temperature since 2009.

While both free BMAA and AEG were detected only up to July 1 (in six out to 19 samples for BMAA and in three of 19 samples for AEG), free DAB was always observed. However, after acid hydrolysis, all isomers were detected and quantified at higher concentrations. The concentrations of total BMAA in mussels showed a time-dependent increase during the summer of 2009, while they were more stable in oyster. The concentrations of total DAB were similar in the two mollusk matrices, between 3.4–9.7 and 3.3–8.8 µg/g DW in mussel and oyster, respectively. As for BMAA, the concentrations of total AEG in mussels increased between 20 June and 7 September. The higher content of BMAA and isomers found in mussels may be explained by their generally higher filtration activity compared to oysters [35].

During the summer, 2009, two phytoplankton blooms dominated by diatoms (e.g., *Chaetoceros* sp.) were observed at the beginning of July and at the end of August in the Thau Lagoon [60]. As diatoms were recently suggested to produce BMAA [30], the occurrence of such microalgae in addition to picocyanobacteria [35] could explain the presence of BMAA in mollusks during the summer, 2009. However, the production of BMAA by microalgae of the Thau Lagoon has not been studied so far.

**Figure 9:**
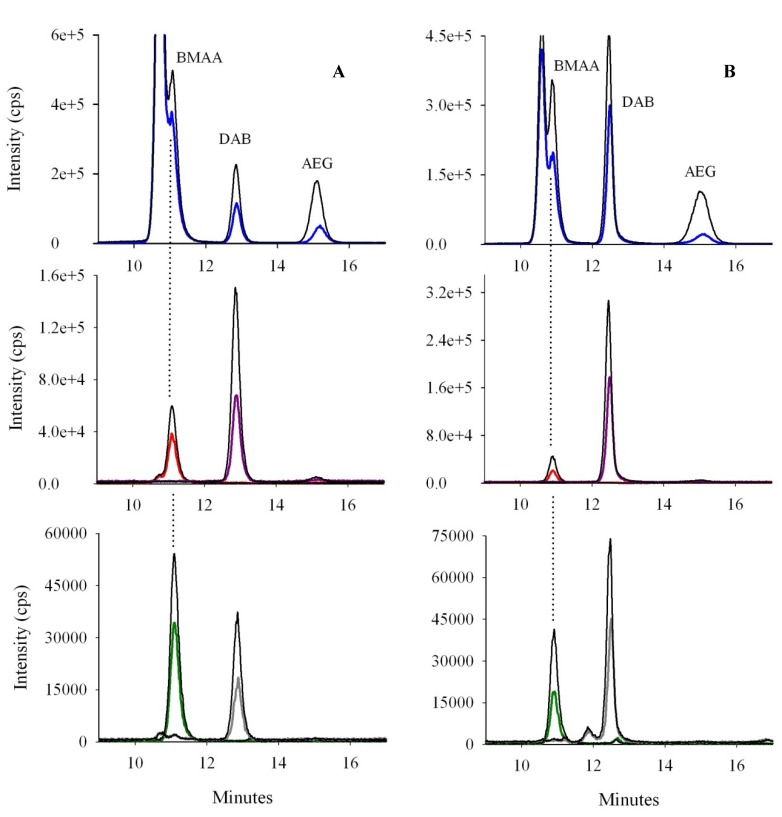
Chromatograms of spiked (dark line) and non-spiked, naturally-contaminated (**A**) oyster and (**B**) mussel samples after performing the extraction for the determination of total BMAA and isomers. Colored lines represent mass spectral transitions *m/z* 119 to *m/z* 102 (blue, top graphs), 88 (red) and 101 (purple) in the middle graphs and 76 (green) and 74 (grey) in the bottom graphs.

As no uncontaminated mussel or oyster samples could be found in the study area, we decided to spike the analytes into extracts of naturally contaminated samples. This exercise has allowed for the verification of retention times and ion ratios. No differences in retention time or ion ratios were detected between naturally-contaminated and spiked samples (Figure 9). It should be noted that after acid hydrolysis, an interfering compound giving a signal at the transition *m/z* 119 > 102 (corresponding to the loss of the NH_3_ group [20]) and eluting just before BMAA was observed for mollusk matrices (Figure 9). This unknown interference always eluted before BMAA, and the retention times of both compounds were reproducible. For example, mean retention times of 10.62 min ± 0.04 and 10.90 min ± 0.04, respectively, for the interference and BMAA in mussel matrix were obtained for seven replicates of the same sample injected twice and one replicate of four other mussel samples.

Similarly, an interfering compound, partially resolved from BMAA, was reported by Jiang *et al.* [27] for cyanobacteria and mollusks from the Baltic Sea, after the derivatization of analytes. Nevertheless, retention time, specific mass spectral transitions (*m/z* 119 to *m/z* 88 and 76) and ion ratios (88/119 and 76/119) allowed us to unambiguously identify BMAA in hydrolyzed mollusk matrices. Moreover, the chromatograms of spiked oyster and mussel matrix clearly showed that the areas of mass spectral transitions corresponding to BMAA (and other isomers) are higher than in the non-spiked samples (Figure 9), thereby confirming that BMAA, DAB and AEG were present in these matrices. This compound could potentially interfere with the quantification of BMAA. However, as no interfering compound was detected with the mass spectral transitions *m/z* 119 to *m/z* 88 and 76, we quantified BMAA in parallel using either the highest transition (which has the interfering compound eluting at the front) or the two specific smaller transitions, and the concentrations obtained with either calculation method were within an error range of 10%–15%. As this error is in the range of the overall method variability, we decided to report quantification with the same transition as for the other isomers for the purpose of consistency.

The presence of BMAA isomers (DAB and AEG in this study plus BAMA in another study [27]) and interfering compounds within the same samples demonstrates the need for highly selective methods to accurately quantify those molecules in biological matrices.

Our results confirmed the presence of BMAA and isomers in mollusks of the Thau Lagoon, but with a HILIC-MS/MS method without the derivatization of samples. The concentrations we obtained here are similar to those that Masseret *et al.* have reported [35] for total BMAA in mussels, but higher for total DAB in mussels and both total BMAA and DAB in oysters. However, the application of a corrective factor is not mentioned in their study.

Apart from Thau Lagoon, BMAA and isomers were also reported in other ecosystems, after the derivatization of samples. If we consider the most selective methods that have been employed, BMAA was severally detected in mollusks coming from the Baltic Sea [12], in mussels and oysters either in very low [13] or at similar concentrations than what we obtained (0.27–1.16 µg/g wet weight corresponding to about 1.3–5.8 µg/g DW of mussel, if wet mussels are assumed to contain 80% water [54]). BMAA was also reported in oysters originating from the southeastern United States (6.8–46.9 µg/g DW) [53], in mollusks of Lake Taihu, China (mean of 3.21 µg/g DW) [15] and in cockles collected on Portuguese coasts (up to 0.434 µg/g DW) [32]. Finally, the presence of BMAA in mollusks of South Florida waters should be considered despite the use of a non-selective LC-FLD method [14].

Concerning BMAA isomers, BAMA and DAB in Swedish oysters [12,27], DAB in blue crabs [59] and DAB and AEG in Portuguese cockles [32] were observed. Interestingly, Lage *et al.* [32] mentioned that “these two isomers were observed along with BMAA in nearly all samples”. Masseret *et al.* [35] also found both BMAA and DAB in all mollusks samples of the Thau Lagoon. When BMAA isomers are considered, they are frequently detected in filter-feeding shellfish mollusk matrices. Recently, they were also reported in shark cartilage dietary supplements with both the FLD and MS/MS detection methods after the derivatization of samples [61].

Nevertheless, as far as we know, this is the first time that BMAA and isomers have been reported in mollusk matrices without the derivatization of samples, suggesting that HILIC-MS/MS methods are suitable to quantify these analytes in complex matrices.

While BMAA toxicity has been well studied [2] and DAB was reported to be a neurotoxic non-proteinogenic amino acid [62], the toxicity of AEG is unknown [25]. The incorporation of BMAA into proteins remains to be “indisputably proven” [63], especially because it is suggested to be involved in BMAA bioaccumulation [5]. Further work is required to understand the bioaccumulation pathway of BMAA in higher trophic levels of ecosystems, as observed in Guam or in the Baltic Sea.

## 3. Experimental Section

### 3.1. Chemicals and Reagents

β-*N*-methylamino-l-alanine hydrochloride (BMAA, B107) and trichloroacetic acid (TCA, 33731) were purchased from Sigma-Aldrich, France, while N-2-aminoethylglycine (AEG, A1153) and 2,4-diaminobutyric acid dihydrochloride (DAB, D0083) were obtained from TCI, Belgium. d-2,4-diaminobutyric acid-2,3,3,4,4-^2^D_5_ dihydrochloride (D_5_DAB), used as the internal standard, was purchased from CDN isotopes (CIL, Sainte-Foy-La-Grande, France).

Methanol (MeOH) and acetonitrile (ACN) were obtained as HPLC grade solvents from JT Baker. Water for analysis was supplied by a Milli-Q integral 3 system (Millipore). Solutions of formic acid (FA, 33015), hydrochloric acid 37% (HCl, 258148) and ammonium hydroxide (221228), all reagent grade, were purchased from Sigma-Aldrich.

Calibrant stock solutions (10 µg/mL) of BMAA, DAB, AEG and D_5_DAB were prepared in Milli-Q water and stored at 4 °C. As observed by Combes *et al.*, 2013 [24], no degradation of stock solutions was observed after more than 6 months in this storage condition.

### 3.2. Samples

#### 3.2.1. Cultures of Cyanobacteria

Ten non-axenic species of cyanobacteria, six of which are reported to be BMAA producers, were cultured for the screening of BMAA and isomers. *Leptolyngbya* PCC 73110 and *Synechocystis* PCC 6803 were maintained in BG11 medium [64]. *Nostoc* PCC 7120 and PCC 7107 were cultured in BG11_0_ (nitrogen free). *Microcystis* PCC 7806 was cultured in modified BG11, BG11_0_ with the addition of 2 mM NaNO_3_ and 10 mM NaHCO_3_ , and *Symploca* PCC 8002 in a mixture of BG11 and modified L1 medium, salinity adjusted to 27, no vitamins added (1:12, v/v). *Nostoc* CCMP 2511 was cultured in modified L1 medium, with four-times the classic NaNO_3_ concentration of L1 medium [65]. *Synechococcus elongatus* CCAP1479/1B was grown in Conway 10%, seawater diluted 3.33-times with Milli-Q water before adding the Conway elements [66] without silica. *Calothrix crustacea* CCAP1410/9 was cultured in F/2 medium [67] and *Nostoc endophytum* CCAP1453/14 in Conway medium (without silica).

The Milli-Q water used to prepare BG11 and modified BG11 medium was supplied by a Milli-Q integral 3 system (Millipore, Saint-Quentin-Yvelines, France), while filter-sterilized seawater at a salinity of 35 was used for seawater culture media. All cultures were maintained under sterile conditions at 22 °C under a 16:8 h light/dark cycle at a light intensity of 50–60 μmol/m^2^/s, except *Synechococcus elongatus*, *Calothrix crustacean* and *Nostoc endophytum* (20 °C, 12:12 h light/dark cycle, light intensity of 300 μmol/m^2^/s). Batch cultures were grown to establish growth factors and toxin production for the strains *Leptolyngbya* PCC 73110 and *Nostoc* CCMP 2511. Biomass was regularly monitored by the measure of DO750 nm (Jenway spectrophotometer 6705 series), and cyanobacteria were sampled after a growth period of 7, 13, 22, 40, 55, 83 and 111 days. All other strains were maintained in batch culture. Cyanobacterial cells were harvested via centrifugation at 4000× *g* for 30 min at 4 °C. The supernatant was carefully discarded, and the resulting pellet was freeze-dried, homogenized and stored at room temperature until further processing.

#### 3.2.2. Collection of Field Samples

During summer, 2009, mussels (*Mytilus galloprovincialis*) and oysters (*Crassostrea gigas*) were sampled in the Mediterranean Lagoon of Thau, France, from two sites (Bouzigues and Marseillan; see Pernet *et al.* [60]). Whole flesh aliquots were freeze-dried, stored at room temperature and homogenized with a Microtron MB 550™ blender (Kinematica) before extraction.

### 3.3. Sample Preparation

#### 3.3.1. Extraction

Aliquots of freeze-dried material (10 mg for cyanobacteria and 15 mg for mollusks) were weighted in a 1.5 mL Eppendorf^®^ tube, and both 0.5 g of 200 µm glass beads and 750 µL of trichloroacetic acid (TCA) 0.1 M were added. Extraction was performed with a mixer mill (Retsch MM400, Germany) for 30 min at 30 Hz. Deuterated DAB (D_5_DAB, concentrations of 30 or 50 ng/mL for the extraction of free and total analytes) was added before extraction as an internal standard. Tubes were centrifuged at 13,000× *g* after grinding to precipitate beads and debris.

For the extraction of free BMAA and isomers, the supernatant was collected and filtered through a 0.22-µm filter (Nanosep^®^ MF, Pall, Mexico) to remove particulate material. The volume was adjusted to 1 mL with TCA 0.1 M before SPE clean-up.

For the extraction of total analytes, the supernatant was collected after grinding and evaporated to dryness under a stream of nitrogen. The residue was dissolved in 600 µL HCl 6 M. Acid hydrolysis was performed in a Thermomixer^®^ comfort (Eppendorf, Fisher Scientific, Illkirch, France) for 24 h at 99 °C to release the bound form of BMAA and isomers from proteins. HCl was dried under a stream of nitrogen, and the residue was dissolved in 1 mL of TCA 0.1 M before SPE clean-up.

#### 3.3.2. SPE Clean-Up

Two kinds of polymeric cation-exchange sorbents (60 mg/3cc cartridges), Bond Elut^®^ Plexa PCX (Agilent Technologies, VWR, France) and Oasis^®^ MCX (Waters, France), were compared with the recoveries of standards. Then, SPE clean-up with the Plexa PCX cartridge was carried out according to Combes *et al.* [24] with some modifications. The mixed-mode sorbents were conditioned with 2 mL of methanol (MeOH) followed by 1 mL of TCA 0.1 M. Then, 1 mL of sample was loaded onto the cartridge, which was consecutively rinsed with 1 mL of HCl 0.1 M and 2 mL of MeOH. After allowing the cartridges to dry, analytes were eluted with 3 + 1 mL of MeOH/NH_4_OH (93:7, v/v). The eluate was evaporated to dryness under a stream of nitrogen at 40 °C. Finally, the residue was dissolved in a mixture of ACN/water (63:37, v/v), both containing 0.1% FA, before injection.

### 3.4. Analysis by LC-MS/MS 

Analysis by liquid chromatography coupled to tandem mass spectrometry (LC-MS/MS) was performed on a Ultra Fast Liquid Chromatograhy (UFLC) (model Nexera, Shimadzu, Champs-sur-Marne, France) coupled to a triple-quadrupole mass spectrometer (5500 QTRAP, AB Sciex). Chromatography was performed with a ZIC^®^-HILIC column (150 × 2.1 mm, 5 μm, Merck Sequant^®^, Fontenay-sous-Bois, France) and a TSK gel amide 80 guard column (2 × 10 mm, 5 μm).

Mobile phases were Milli-Q water (Mobile Phase A) and ACN (Mobile Phase B), both containing 0.1% formic acid. The flow rate was 0.2 mL/min, and the injection volume was 5 μL. The column temperature was 30 °C, while samples were kept at 4 °C. The linear gradient elution started with 37% of Mobile Phase A, rising to 55% over 18 min, held for 2 min, then decreased to 37% of Mobile Phase A for 3 min and held for 15 min to equilibrate the system. In order to reduce the quantity of impurities entering the MS system, the flow of the first five minutes was directed to the waste container.

To optimize the chromatographic resolution of BMAA and its isomers, a gradient elution was developed thanks to a 2^3^ factorial design. Resolution was calculated as 
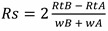
 with *Rt* the retention time and *w* the width at the base of the common mass spectral transition *m/z* 119 > 102. Results were analyzed and interpreted using STATGRAPHICS Centurion XV software.

The LC-MS/MS system was used in positive ion mode with multiple reaction monitoring (MRM) detection. The most intense and common transition *m/z* 119 > 102 was used to quantify BMAA, DAB and AEG, while the transition *m/z* 124 > 47 was used to quantify D_5_DAB, because the other transitions (e.g., *m/z* 124 > 106) suffered from a high background signal.

To increase selectivity, two specific mass spectral transitions of BMAA (*m/z* 119 > 88 and 119 > 76) and DAB (*m/z* 119 > 101 and 119 > 74) and the ion ratios 88/102 and 76/102 for BMAA and 101/102 and 74/102 for DAB were also monitored. A sample was considered positive if the specific transitions were detectable and the variability of ion ratios observed in samples was less than 20%. Classically, the accepted variability is between 10% and 20% for ratios of this height of order. As we observed differences of less 10% for all analytes, we obtained an acceptable variability of ion ratios between standards and samples.

The ESI interface was operated using the following parameters: curtain gas 20 psi; temperature 600 °C; Gas 1 40 psi; Gas 2 60 psi; ion spray voltage 5500 V. For detection, the parameters are reported in Table 4.

BMAA and isomers were quantified using a five-point calibration curve of pure standards constructed by dilution series (1–100 ng/mL) of stock solutions in a mixture of ACN and water (63:37, v/v, both containing 0.1% FA). A corrective factor derived from D_5_DAB recovery was applied to quantify more accurately BMAA, DAB and AEG. The software Analyst 1.5.1 was used to analyze the acquired data.

**Table 4:** Parameters used for optimal detection of BMAA and isomers with the 5500 QTRAP mass spectrometer.

Transition (*m/z*)	EP (V)	DP (V)	CE (eV)	CXP (V)
**119 > 102**	10	81	13	12
**124 > 47**		71	21	22
**119 > 88**		66	17	10
**119 > 76**		66	17	10
**119 > 101**		86	11	8
**119 > 74**		86	19	8

EP, entrance potential; DP, declustering potential; CE, collision energy; CXP, collision cell exit potential.

### 3.5. Recoveries and Matrix Effects

Recoveries of the SPE clean-up step and remaining matrix effects were estimated with one cyanobacterial and two shellfish matrices after extraction of both free and total BMAA and isomers. It has to be noted that no clean matrices were found (particularly after acid hydrolysis). As a compromise, a naturally-contaminated matrix was analyzed in parallel with and without spiking, and the peak areas of the non-spiked samples were subtracted from the peak areas of the spiked samples.

The *Leptolyngbya* PCC73110 (grown 40 days in conditions reported in Section 3.2.1) was chosen as the cyanobacterial matrix. The two shellfish matrices, mussel (*M. galloprovincialis*) and oyster (*C. gigas*), were sampled in 2009 in the Thau Lagoon where BMAA and DAB had been reported [35].

Recoveries of the SPE clean-up protocol with the Plexa PCX cartridge were evaluated with these three matrices with the extraction of both free and total analytes (see Section 2.2.1). Matrices were spiked in triplicate just before SPE with a mixture of BMAA and isomers at a final concentration of 30 ng/mL.

The remaining matrix effects were also assessed for both free and total extraction procedures. Before injection, the dried matrices subjected to extraction procedures were dissolved in a spiked ACN/water mixture to establish three-point standard curves (3, 10, 50 ng/mL). The slope of the spiked matrix curves was compared to the slope of standard curves in solvent to estimate the matrix effects.

### 3.6. Statistical Analysis

Statistical analyses were carried out with SigmaPlot 12.5 (Systat Software Inc., Chicago, IL, USA).

## 4. Conclusions

We present here a highly selective HILIC-MS/MS method to confidently identify and accurately quantify underivatized BMAA and isomers (DAB and AEG) in cyanobacterial and shellfish samples, in both free and total form. Thanks to an optimized gradient elution and an effective SPE clean-up step, good sensitivity (LOQ of 0.22 and 0.15 µg/g DW for cyanobacteria and mollusks, respectively) and low matrix effects were obtained. The use of D_5_DAB as the internal standard allowed for correction and accurate quantification of BMAA and isomers in all samples. This reliable method was applied to screen ten species of cyanobacteria. Neither free nor bound BMAA were found in our cultures. Nevertheless, DAB, a neurotoxic isomer of BMAA, was commonly detected. The HILIC-MS/MS method was also used to screen mollusks collected in 2009 in the Thau Lagoon. BMAA was identified in all shellfish samples after acid hydrolysis, and the concentrations of total BMAA were similar to those reported by Masseret *et al.* [35] in mussels. Additionally, we identified free BMAA in some samples during the month of June, 2009; however, no free BMAA was detected in either mussels or oysters after July 1, 2009. The significance of the general occurrence of DAB and to a lesser extent AEG in different matrices (cyanobacteria and mollusks) should be assessed further. Research is currently underway in our laboratory to pinpoint phytoplankton organisms producing BMAA in the Thau Lagoon. Furthermore, we intend to study the spatio-temporal distribution of BMAA and isomers over a longer time period and in other areas to provide data for risk evaluation.
